# Estimating the degree to which distance and temperature differences drive changes in fish community composition over time in the upper Mississippi River

**DOI:** 10.1371/journal.pone.0225630

**Published:** 2019-12-02

**Authors:** James H. Larson, Jon M. Vallazza, Brent C. Knights

**Affiliations:** U.S. Geological Survey, Upper Midwest Environmental Sciences Center, La Crosse, WI, United States of America; University of Maine at Farmington, UNITED STATES

## Abstract

Similarity in community composition declines as distance between locations increases, a phenomenon that has been observed in a wide variety of freshwater, marine and terrestrial ecosystems. One driver of the distance-similarity relationship is the presence of environmental gradients that alter the suitability of sites for particular species. Although some environmental gradients, such as geology, do not change on a year-to-year basis, others, such as temperature, vary annually and over longer time periods. Here, we used a 21-year dataset of fish communities in the upper Mississippi River to examine the effect of distance on variation in community composition and to assess whether the effect of distance is primarily due to its effect on thermal regime. Because the Mississippi River is aligned mostly north-to-south, larger distances along the river roughly correspond to larger differences in latitude and therefore thermal regime. As expected, there was a moderate distance-similarity relationship, suggesting greater distance leads to less similarity. The effect of distance appeared to increase slightly over time. Using a subset of data for which air temperature was available, we compared models that incorporated both difference among sites in degree days (a surrogate for thermal regime) and physical distance (river km). Although physical distance presumably incorporates more environmental gradients than just temperature (and other potential mechanisms), temperature alone appears to be more strongly associated with differences in the Mississippi River fish community than distance.

## Introduction

Similarity in community composition between two sites has a tendency to decrease as the physical distance between the sites increases. This appears to be a general phenomenon of freshwater, marine and terrestrial ecosystems [1]. Soininen et al. [1] identified 3 main mechanisms that account for the relationship between distance and similarity: 1) decreasing similarity in environmental features, 2) increasing likelihood of dispersal barriers between sites, and 3) random fluctuations in abundance and occurrence due to biota’s limited dispersal ability. The first two mechanisms suggest that the relationship between community composition and distance will change over time, either as barriers to dispersal are constructed or removed [2,3] or as environmental conditions change [4]. In cases where environmental gradients drive differences between two communities, changes in the magnitude of the environmental gradient could be related to changes in the magnitude of the similarities between those communities.

Variation in thermal regime is linked to many aspects of ecosystem structure and function due to its fundamental influence on metabolic processes [5,6]. In aquatic ecosystems, variation in temperature is also linked to the growth and survival of many species [7,8], and changing temperature appears capable of altering community composition in at least some taxa [9]. Temperature is therefore an environmental gradient that varies through time that could cause a distance-similarity relationship.

Chick et al. [10], found a very strong relationship between distance and similarity within the fish community of the Mississippi River. The Mississippi River is generally oriented north-to-south, so as distances increase, so do differences in latitude, and some species are only abundant in northern or southern sites (e.g., Yellow Perch in northern sites). Therefore, temperature differences across sites in the Mississippi River are possibly important in creating the distance-similarity relationship, and these gradients in temperature change annually. Furthermore, longer-term trends in environmental conditions may have increased or decreased the similarity of these communities. For example, if increasing temperatures over the past 20 years have reduced the abundance of northern species (e.g., Yellow Perch), then the northern community may become more similar to the southern one. Few long-term datasets exist that can be used to examine how distance-similarity relationships vary over time, especially in river systems. In the upper Mississippi River, the U.S. Army Corps of Engineers’ Upper Mississippi River Restoration Program, Long Term Resource Monitoring element (UMRR LTRM) has collected data on fish community composition using standardized methods since 1991. Here, we use fish community composition data collected by the LTRM to answer three questions: 1) What is the magnitude of the distance-similarity relationship in the upper Mississippi River fish community? 2) Is the distance effect increasing or decreasing over time? and 3) Is temperature an important environment gradient causing the distance-similarity relationship?

## Methods

### Ethics statement

No new biological data were collected for this study.

### Study sites

The upper Mississippi River (UMR) is broken into a series of navigation pools by 29 dams numbered from north to south (i.e., Dam 1 is upstream of Dam 2). Some dams potentially restrict fish passage depending on dam operation and fish biology [11]. During high discharge, the adjustable gates on most of these dams are lifted completely out of the water. In such conditions (termed “open river”), migratory fishes move more easily upstream (e.g., [12–14]). Only two dams likely act as barriers to fish passage year-round because of lift heights that exceed 11 m: upper St. Anthony Falls near St. Paul Minnesota (with locks now permanently closed to prevent Asian carp upstream movement) and Dam 19 near Keokuk, Iowa [11]. All of these dams have been in place since before 1950 (i.e., they predate all of the data used in this study by several decades). For Dam 19, fish movement upstream is limited to the navigation lock, and some migratory species appear capable of moving through these lock chambers [15].

**Fig 1:**
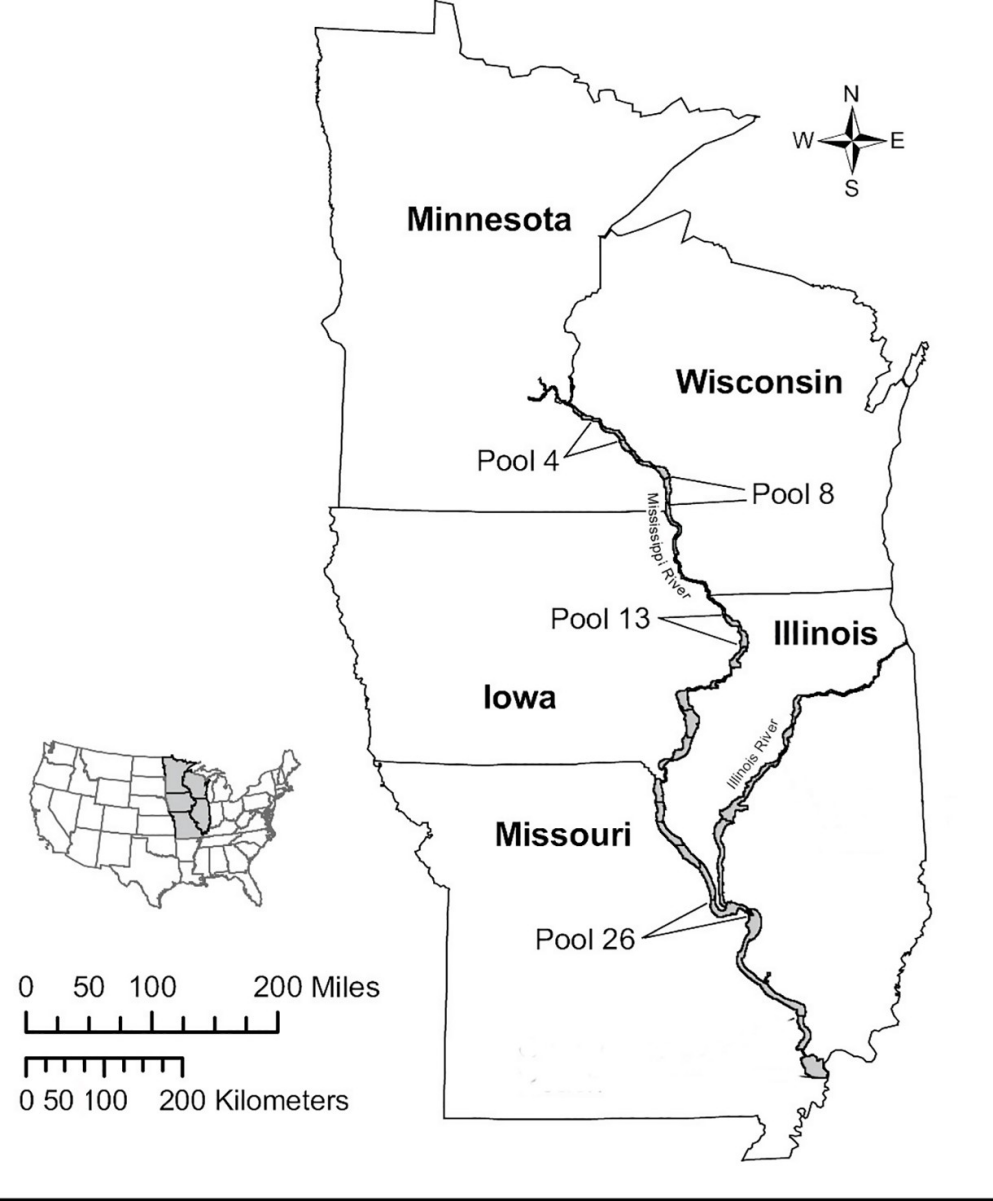
Locations of U.S. Army Corps of Engineers’ Upper Mississippi River Restoration, Long Term Resource Monitoring element sampling locations within the upper Mississippi River that are used in this study. Original map appears in [17].

The UMRR LTRM performs standardized annual sampling on four pools in the UMR (Fig 1): Pool 4 (river km 1212–1283), Pool 8 (1093–1131), Pool 13 (841–896) and Pool 26 (327–389). The UMRR LTRM conduct standardized sampling of the La Grange Pool of the Illinois River and the Open River reach of the Mississippi River, these sites were excluded from our analysis because of their distinct geomorphology, lack of locks and dams, and, for the La Grange Pool, because it was outside the Mississippi River proper. Each pool is divided into four aquatic habitats: main channel, side channels, backwaters and impounded areas [16].

### Fish sampling

Fish data are available from the UMRR LTRM at https://www.umesc.usgs.gov/ltrmp.html. The UMRR LTRM uses several gears to collect fish, but in this study we will focus solely on the daytime electrofishing data [16]. Daytime electrofishing methods used by the UMRR LTRM are described elsewhere [16]. Briefly, electrofishing runs last approximately 15 minutes using standardized power. Sampling locations are randomly selected within an aquatic habitat type. These methods have been used to collect fish community data since 1993. We used data from 1993–2014 (21 years) for this analysis. Our analysis was limited to samples collected from main channel border habitats because other habitats are sometimes absent from individual pools or have much greater variability in catchability (based on personal observation). We used two different species lists to model differences in community composition: one that uses all the available data and one that includes only species that make up at least 1% of the catch in at least one pool (S1 Table). This was because not all species that occur in the data set are likely to be well-represented by the sampling methods used here. For example, some species occur in the dataset that are unlikely to be residents of the Mississippi River (e.g., Brown Trout, *Salmo trutta*) and other species are poorly sampled by electrofishing (e.g., Paddlefish, *Polyodon spathula*).

### Temperature data

Water temperature data were not available, so instead we used air temperatures to estimate differences in temperatures between sites. All data were obtained from the National Oceanic and Atmospheric Administration's National Climatic Data Center (http://www.ncdc.noaa.gov/data-access; Pool 4, USC00470124; Pool 8, USW00014920; Pool 13, USC00130608; Pool 26, USC00237397). Following Chezik et al. [8] the degree days for a single day (DD;°C-days) are calculated as:

$$DD=\left[\frac{Tmax+Tmin}{2}\right]-T_{x}$$

T_x_ is the threshold temperature under consideration (e.g., 0°C or 5°C) and T_max_ and T_min_ are the daily maximum and minimum temperatures, respectively. Negative daily DD estimates are discarded, and the positive daily DD estimates are summed over the period of interest. We calculated DDs for a T_x_ of 0°C, 5°C, 10°C, 15°C, 20°C and 25°C, since we were uncertain which (if any) DD thresholds would be best for describing variation in similarity. If air temperature data for more than 5 days were missing, we excluded that year from analysis. For each combination of pools, we calculated the cumulative difference in degree days over the previous 1- or 2-year period, under the assumption that a lag period might strongly influence the fish community (many of which are not young-of-year when captured with the electrofishing gear).

### Statistical analysis

Statistical analyses were performed in R [18] and example code is provided in the S1 File (Note: Any use of trade, product, or firm names is for descriptive purposes only and does not imply endorsement by the U.S. Government). To calculate the magnitude of the distance-similarity relationship, we first calculated similarity among locations using the Bray-Curtis dissimilarity index, as implemented in the R package ‘vegan’ [19,20]. In the Bray-Curtis dissimilarity index, this value incorporates the abundance of individual species and ignores species absent at both sites. For the purpose of making the results easier to understand we report similarity, which is 1-dissimilarity, so higher values indicate more similarity (these calculated dissimilarities are included in the S2 File). We calculated similarity index values for all pool-year combinations using A) each species that made up at least 1 percent of the catch in any pool (22 species total) and B) all species that occurred in any pool (140 species total). Species are listed in S1 Table. All data were square root transformed and standardized (using the ‘Wisconsin’ standardization in the ‘vegan’ package) prior to analysis [20].

To assess how robust our similarity measurements are to small differences in the number of samples collected, we performed resampling on our data at the level of the electrofishing run. For each resampling, 10% of the electrofishing runs were discarded randomly and the Bray-Curtis similarity index was calculated for all site-year combinations using the remaining data. We repeated this resampling procedure 500 times and then calculated mean and standard deviation of the similarity for all site-year combinations (see S1 File). We used the mean values in all the subsequent analyses.

The standard distance-similarity relationship can be estimated using a simple linear regression to relate log-transformed distance to log-transformed similarity [1]. However, we used a multilevel model to estimate the relationship between distance (in river km; log transformed) and Bray-Curtis similarity (log-transformed), where the slope of the distance effect could vary by year (a random effect). This was implemented using the lme4 package in R [21]. We compared this model to a model that included only a year effect, which we treated as a null model, using Akaike’s information criterion, corrected for small sample size (AIC_C_) [22]. We also calculated a ‘marginal’ R^2^ value (following [23]), which is the variation explained by the predictor variables independent of the grouping variable (i.e., the R^2^ of the effect of distance but not year). Prior to analysis, all variables were standardized using by subtracting by the mean and dividing by the standard deviation to create variables where the mean is zero and the standard deviation is 1, so that standardized effects could be calculated (β_DIST_).

To assess whether there was a temporal trend in the effect of distance on similarity, we used a simple linear regression (using base-R lm() function) to relate the year-specific standardized slopes (β_DIST|YEAR_) to the year. Again, this simple linear regression was compared to a null model (a model with only a constant) using AIC_C_ [22], and a conventional R^2^ was calculated.

Finally, 26 models were compared using AIC_C_ to assess the role of temperature relative to physical distance (in river km). The two multi-level models from question 1 (the null model and the model that included distance varying by year) were compared to multi-level models that also included the difference in degree days over the previous 1 or 2 years (at every temperature threshold 0°, 5°, 10°, 15°, 20° and 25°C). We included a 1 or 2 year lag time because many species cannot be effectively sampled using electrofishing in their first 1 or 2 years of life. In all cases these data were standardized so that standardized slopes of the distance (β_DIST_) and degree-day (β_DD_) effect could be calculated. Standardized slopes are preferred for this, since they can be compared despite different units. This multi-model comparison was performed using only data that were available for all variables. In this case, much less temperature data (72 observations) were available than fish data (132 observations).

## Results

### What is the magnitude of the distance-similarity relationship in the Mississippi River fish community?

**Fig 2:**
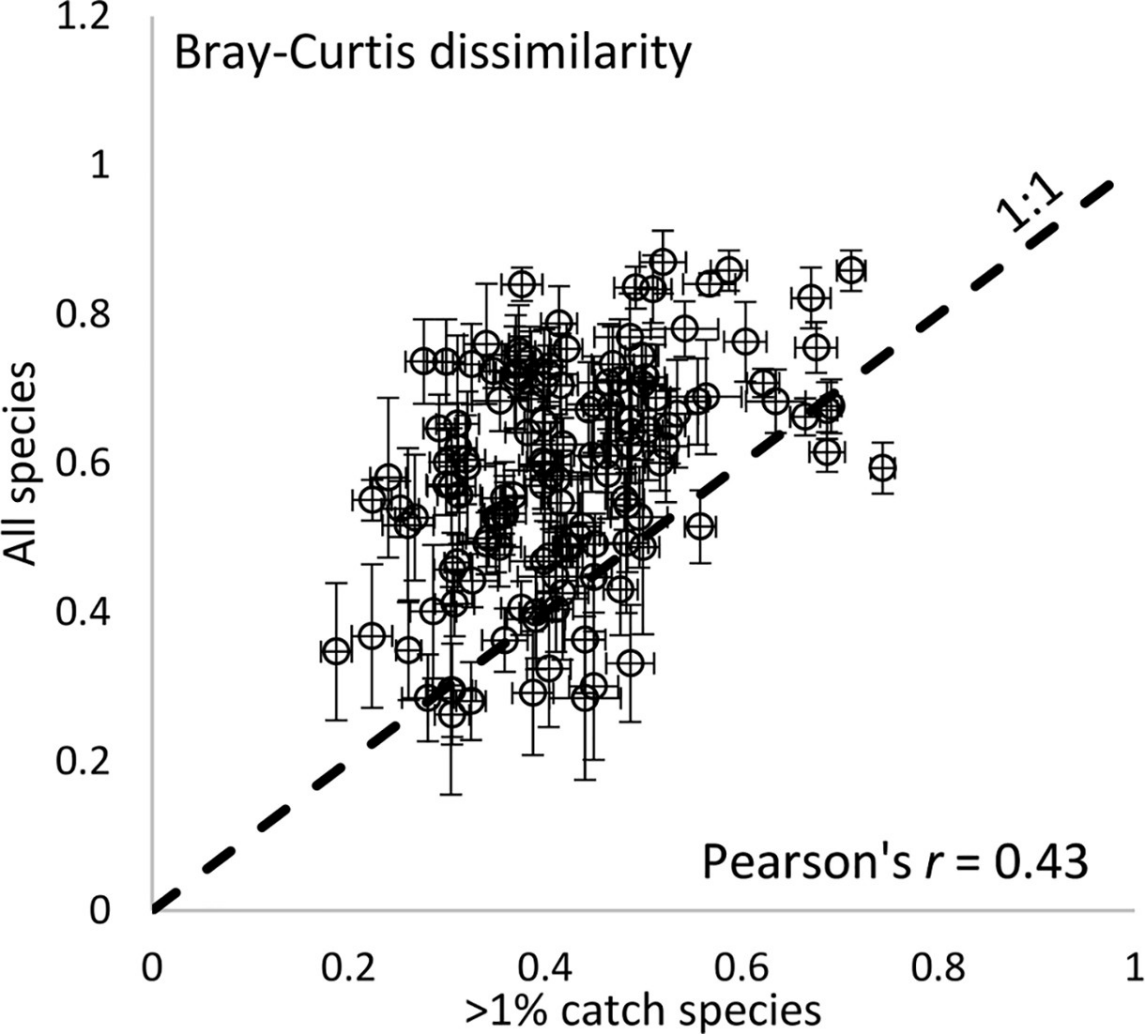
Association between Bray-Curtis similarity estimated using two different species lists. One list includes 22 species that make up at least 1% of the catch in at least one pool (≥1% catch species) and the other list includes all species that occur in at least one pool. Each dot is the difference between two pools in a single year (overall averages are reported in Table 1). Error bars are the standard deviation estimated by resampling.

The species list used had a large effect on Bray-Curtis similarity (Table 1). Pools showed lower similarities when all species were included in the analysis than when only the ≥1% species were included (Table 1, Fig 2). Similarity calculated between the two species lists was not strongly correlated (Pearson’s *r* = 0.43; Fig 2), so the results depended strongly on which species list is being used.

**Table 1:** Average of the Bray-Curtis similarity index from 1993–2014 between pools in the upper Mississippi River. The coefficient of variation is given in parentheses and the range is in brackets. Distance is from the middle of each pool (by river kilometers).

Site comparison	Similarity (≥1% species list)	Similarity (all species)	Distance (river km)
Pool 4, Pool 8	0.66 (11.8) [0.46,0.78]	0.48 (25.0) [0.22, 0.74]	135.6
Pool 8, Pool 13	0.60 (12.7) [0.47,0.74]	0.49 (24.5) [0.28, 0.70]	243.0
Pool 4, Pool 13	0.65 (10.7) [0.51,0.81]	0.43 (37.2) [0.16, 0.72]	378.6
Pool 13, Pool 26	0.60 (11.6) [0.44,0.72]	0.44 (36.4) [0.27, 0.71]	511.0
Pool 8, Pool 26	0.42 (22.5) [0.26,0.62]	0.30 (33.3) [0.13, 0.49]	754.0
Pool 4, Pool 26	0.53 (13.8) [0.31,0.65]	0.34 (35.3) [0.14, 0.57]	889.6

The range of observed Bray-Curtis similarities over the 21-year period within a particular pool-pool combination was considerable. For example, when only the 22 species that made up ≥1% of the catch were included, the similarity between Pools 4 and 8 (the closest sites in the dataset at 135.6 river km) ranged between 0.46–0.78 (Table 1). This overlaps considerably with the similarities between Pools 4 and 13 (0.51–0.81) despite much greater separation distance (378.6 river km; Table 1).

**Fig 3:**
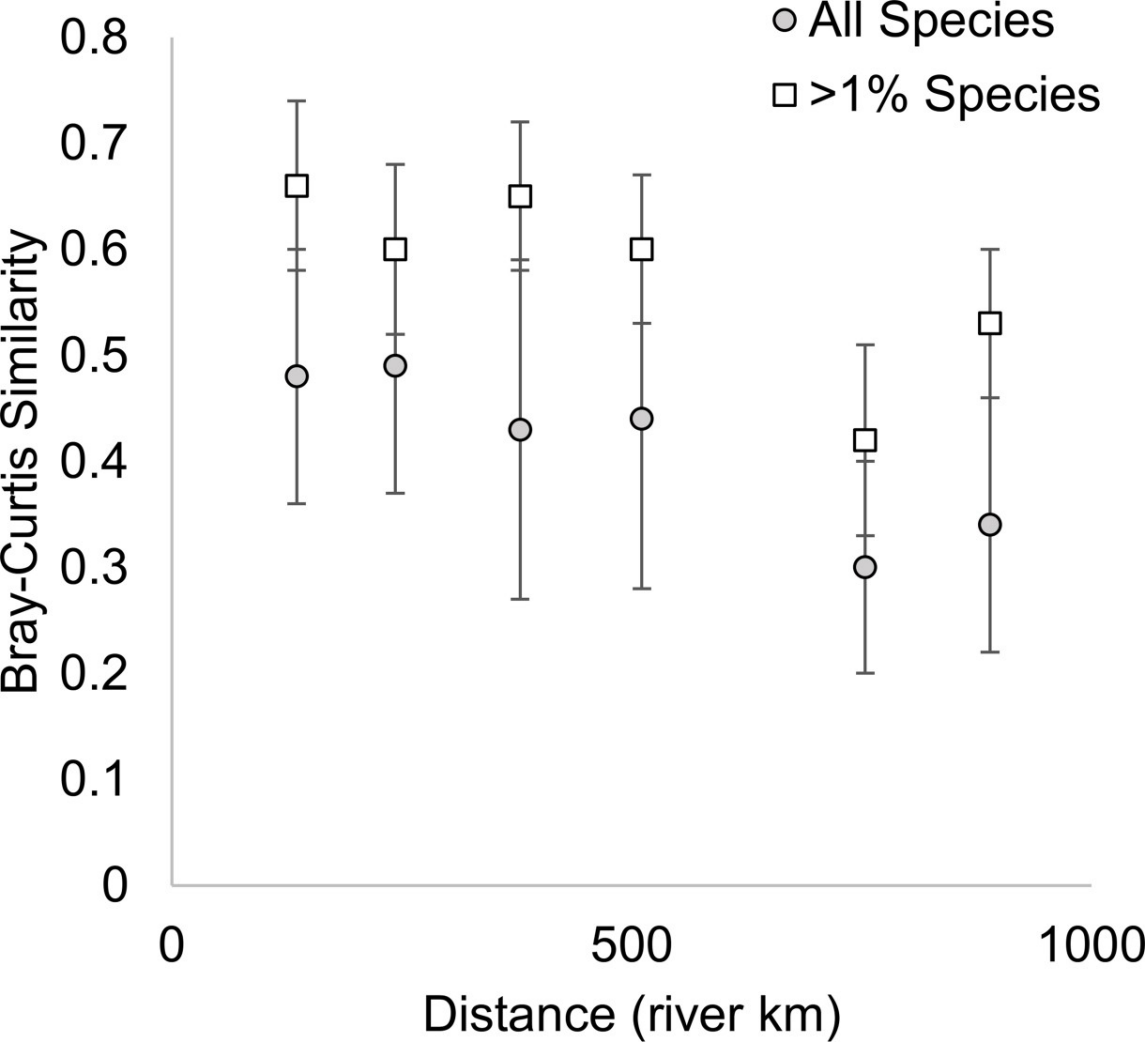
Average similarity (with standard deviation) over the 21 years for which data are available between fish communities of pools of the upper Mississippi River (Pools 4, 8, 13 and 26).

Using the ≥1% species list, distance (river km) had a moderate effect on similarity (β_DIST_ = -0.53, 95% confidence interval [-0.67,-0.38]) and distance explained a moderate amount of variation in similarity (marginal R^2^ = 0.28; Fig 3). This multi-level model had an AIC_C_ value that was ~42 units lower than a comparable null model. The effect of distance was less when all species were used to calculate similarity (β_DIST_ = -0.42, [-0.56,-0.28]) and distance explained less variation in similarity (marginal R^2^ = 0.18). The multi-level model using all species had an AIC_C_ value that was ~28 units lower than a comparable null model. Since the all species list includes species that are poorly sampled with these methods, we have opted to set it aside for the following questions.

### Is the distance effect increasing or decreasing over time?

**Fig 4:**
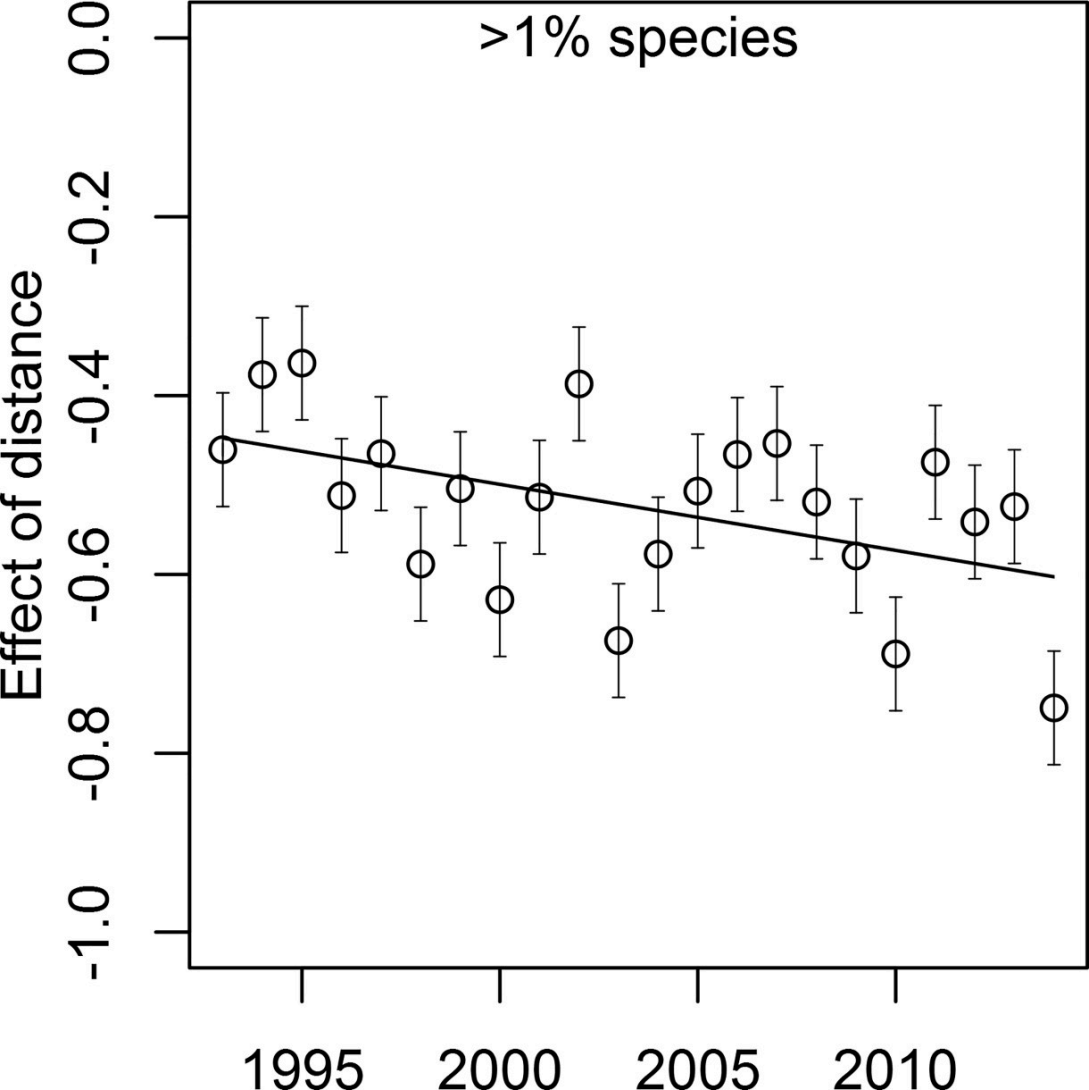
Magnitude of the standardized slope (the effect) relating distance (river km) to Bray-Curtis similarity over time. Estimated effect sizes (slopes) are derived from a multi-level model where slope was allowed to vary by year. Only species that made up at least 1% of the catch in at least one pool were included in this analysis.

In the multi-level model, the distance effect could vary by year, and it was therefore possible to identify years where the association between distance and similarity was stronger or weaker. For the ≥1% species list, β_DIST_|_YEAR_ varied between -0.36 and -0.75 (Fig 4). A simple linear regression relating β_DIST_|_YEAR_ to year had an AIC_C_ that was ~3 units lower than a comparable null model and the standardized slope was negative (*b* = -0.48 [-0.89,-0.07], R^2^ = 0.16), suggesting that the effect of distance was slightly increasing over time.

### Is temperature an important environment gradient causing the distance-similarity relationship?

Air temperature data for Pool 13 and Pool 26 contained gaps that prevented us from calculating the difference in degree days for several years (11 of the 21 years for Pool 26). Simple Pearson’s correlation coefficients between degree days and year are always positive and vary in magnitude depending on the individual pool and threshold temperature. Overall, degree days seem to be increasing the most in Pool 13 and Pool 26 (although sample sizes are also smaller for these pools; Table 2). In three of the four pools, the biggest change is in the DD15.

**Table 2:** Pearson’s correlation coefficients with 95% confidence intervals between year and degree days of air temperature. Sample size for Pools 4 and 8 is 22. Sample size for Pool 13 is 20. Sample size for Pool 26 is 11. **Bold** indicates a 95% confidence interval that does not overlap zero.

Variable	Pool 4	Pool 8	Pool 13	Pool 26
Degree days >0° C	0.39 [-0.18,0.61]	0.10 [-0.34,0.50]	**0.46 [0.03,0.75]**	0.49 [-0.15,0.84]
Degree days >5° C	0.33 [-0.11,0.66]	0.14 [-0.30,0.53]	**0.54 [0.13,0.79]**	0.53 [-0.10,0.86]
Degree days >10° C	0.39 [-0.03,0.70]	0.17 [-0.27,0.55]	**0.59 [0.20,0.82]**	0.57 [-0.05,0.87]
Degree days >15° C	**0.42 [0.001,0.72]**	0.19 [-0.25,0.57]	**0.57 [0.17,0.81]**	**0.62 [0.03,0.89]**
Degree days >20° C	0.35 [-0.09,0.67]	0.11 [-0.33,0.51]	0.42 [-0.03,0.73]	0.54 [-0.09,0.86]
Degree days >25° C	0.28 [-0.16,0.63]	0.09 [-0.35,0.49]	0.22 [-0.24,0.61]	0.42 [-0.24,0.82]

Models incorporating both temperature and physical distance had a more limited sample size (72 observations) than models reported for question 1 (above; 132 observations) due to missing temperature data for many pools. Still, a model that includes degree days and distance is more strongly supported than the model that includes only distance (compare model 5 to model 3 in Table 3). Models with only the difference in degree days above zero over the previous 1 or 2 years (e.g., model 1 and 2) are also an improvement over the distance model (model 5; Table 3). Physical distance (river km) and difference in degree days are strongly correlated (Pearson’s r >0.7 for all the degree day thresholds), so identifying the unique contribution of distance and temperature is difficult. When both temperature and distance appear in the same model, the effect sizes are lower than when they appear in models with only one predictor variable (Table 3), suggesting both variables are explaining some of the same variation. For example, β_DIST_ is -0.44 in model 5, but β_DIST_ is -0.24 in model 3, which also includes the difference in degree days above 5°C. Overall, these models did not explain most of the variation in pool-to-pool similarity (marginal R^2^ <0.35 in all cases; Table 3).

**Table 3:** Results of model selection procedure. Distance refers to the physical distance (in river km) between pools, while DD refers to the difference in degree days between pools over the previous 1 or 2 years. Threshold temperatures of 0°C, 5°C, 10°C, 15°C, 20°C and 25°C were compared, but only the threshold with the strongest support (assessed with AIC_C_) was parameterized for these purposes (**bolded models**). For example, if a model with DD0 had a lower ΔAIC_C_ than an otherwise identical model with DD5, we only parameterized the model with DD0. B-C Sim = Bray-Curtis similarity; β_DIST_ = standardized slope relating distance to B-C Sim; β_DD_ = standardized slope relating difference in degree days to B-C Sim. Because temperature data are relatively limited, only 72 observations were included in this analysis.

No.	Model	ΔAIC_C_	Marginal R^2^	β_DIST_	β_DD_
**1**	**B-C Sim = 1|Year +DD0 (2 year)**	**0**	**0.29**	**-**	**-0.58 [-0.78,-0.36]**
	B-C Sim = 1|Year +DD5 (2 year)	0.6	0.29		
**2**	**B-C Sim = 1|Year +DD0 (1 year)**	**0.9**	**0.28**	**-**	**-0.57 [-0.77, -0.35]**
	B-C Sim = 1|Year +DD5 (1 year)	1.5	0.27		
	B-C Sim = 1|Year +DD10 (2 year)	2.3	0.27		
**3**	**B-C Sim = Distance + Distance|Year +DD5 (2 years)**	**2.4**	**0.32**	**-0.25 [-0.53, 0.05]**	**-0.33 [-0.61,-0.04]**
	B-C Sim = 1|Year +DD10 (1 year)	2.6	0.26		
	B-C Sim = Distance + Distance|Year +DD0 (2 years)	2.6	0.32		
	B-C Sim = Distance + Distance|Year +DD10 (2 years)	2.9	0.31		
**4**	**B-C Sim = Distance + Distance|Year +DD10 (1 years)**	**2.9**	**0.31**	**-0.28 [-0.55,0.03]**	**-0.30 [-0.57,-0.02]**
	B-C Sim = Distance + Distance|Year +DD5 (1 years)	3.1	0.31		
	B-C Sim = Distance + Distance|Year +DD25 (1 years)	3.1	0.31		
	B-C Sim = Distance + Distance|Year +DD15 (1 years)	3.3	0.30		
	B-C Sim = Distance + Distance|Year +DD0 (1 years)	3.3	0.31		
	B-C Sim = Distance + Distance|Year +DD15 (2 years)	3.6	0.30		
	B-C Sim = Distance + Distance|Year +DD20 (1 years)	3.9	0.29		
	B-C Sim = 1|Year +DD15 (2 year)	4.1	0.25		
	B-C Sim = Distance + Distance|Year +DD20 (2 years)	4.3	0.29		
	B-C Sim = 1|Year +DD15 (1 year)	4.3	0.24		
	B-C Sim = Distance + Distance|Year +DD25 (2 years)	4.9	0.28		
**5**	**B-C Sim = Distance + Distance|Year**	**4.9**	**0.23**	**-0.44 [-0.68,-0.12]**	**-**
	B-C Sim = 1|Year +DD20 (2 year)	6.0	0.23		
	B-C Sim = 1|Year +DD20 (1 year)	6.5	0.22		
	B-C Sim = 1|Year +DD25 (1 year)	7.0	0.21		
	B-C Sim = 1|Year +DD25 (2 year)	7.4	0.21		
6	**B-C Sim = 1|Year**	**19.8**	**-**	**-**	**-**

## Discussion

### What is the magnitude of the distance-similarity relationship in the Mississippi River fish community?

Although distance appears to be fundamentally associated with reduced similarity between sites in community composition [1], it is important to consider carefully how sensitive these relationships are to the species being included. The species included in the measurements of community composition were a major factor dictating how similar two communities appeared in this dataset. Going from the species that made up at least 1% of the total catch (22 species) to all species that occurred in the database (140 species) substantially decreased measured similarity between pools. However, using the complete species list decreased the dependence of those differences on physical distance between sites. Although this could indicate that rare species are less influenced by the mechanisms that cause changes in abundance in more common species, it seems more likely that this is a result of methodological challenges. Many widespread species are not easily captured by electrofishing gear (e.g., Paddlefish, *Polyodon spathula*). Therefore, electrofishing data may not provide meaningful information about their relative abundance [24]. Inclusion of these rarely occurring species reduces the similarity between sites, but with so few observations it is difficult to have confidence that these are plausible estimates of relative abundance. In addition, some of these rare species are stochastic occurrences that aren’t representative of a resident population. For example, species swept into the main stem of the Mississippi River from small tributary streams are sometimes captured even though they are not believed to maintain main-stem populations (e.g., Brown Trout, *Salmo trutta*). For these reasons we focus on the results from the ≥1% species list.

Using species lists similar to our ≥1% list, Chick et al. [10] found that the equivalent of our β_DIST_ was -0.80 and Anderson et al. [25] found β_DIST_ was -0.89 in two separate years. The comparable value obtained from this dataset was -0.55 (using the ≥1% species list). Although this relationship varied from year to year (between -0.36 and -0.75 in the ≥1% species list), it was never as strong as the relationship observed in previous studies that include more sites but just one or two years of data [10,25]. Although these slope estimates seem very different, the differences in the data make direct comparisons difficult. For example, Anderson et al.’s [25] 2014 data contained 55 pool-pool similarities, whereas each year in our data set included only 6 pool-pool similarity estimates.

### Is the distance effect increasing or decreasing over time?

In the long-term dataset used in this study, the effect of distance on variation in fish communities may have been increasing slightly over time. Given the large uncertainties and small effect sizes associated with fish catch data, this trend over time should be viewed with caution. Still, the evidence here suggests the fish community within the Mississippi River is undergoing a change that is increasing the effect of distance.

Analysis of how distance-similarity relationships change over time within the same set of sites is very rare. Araújo et al. [26] evaluated the distance similarity relationship in a large river fish community from South America and compared the relationship before and after the installation of a major impoundment. That study found major changes, which were attributed to the creation of a large dispersal barrier and corresponding new habitat types. Nothing equivalent to that major geomorphological change occurred in the Mississippi River over the time frame studied here. In general, the literature provides very little guidance on how much annual variation to expect over periods without such dramatic alterations to the environmental conditions.

Soininen et al. [1] highlighted three mechanisms that cause the decay in similarity of community over distance: 1) environmental gradients, 2) dispersal barriers and 3) random variation in dispersal. One of the most obvious environmental gradients in the Mississippi River sites is the thermal regime. Global climate patterns have been shifting over the time period of this study [27], so it may be that these climatic changes have created steeper environmental gradients that, in turn, drive a stronger distance-similarity relationship. We found that differences in air temperature degree days (a surrogate for differences in thermal regime among sites) were strongly related to community similarity in the subset of the data for which we could find temperature data (see question below).

The other mechanisms mentioned by Soininen et al. [1] individually probably do not account for the increasing effect of distance over time in the Mississippi River. No new dispersal barriers have appeared in the study area during the study period and random variation in dispersal would be unlikely to change in a particular direction over time. If the existing navigation dam network is a strong dispersal barrier [28], then individual pools may be on different community composition trajectories, which could lead to an apparent increase in the effect of distance. This seems unlikely because active navigation dams are not complete barriers to fish passage [14] and many common species, such as Bluegill (*Lepomis macrochirus*), Largemouth Bass (*Micropterus salmoides*) and Channel Catfish (*Ictalurus punctatus*) can complete their life cycles within a single pool. Previous authors have used distance-similarity relationships to test for the effect of navigation dams and concluded they have little effect [10]. Dispersal barriers seem most likely to influence species that require long migrations to complete their life-cycle (e.g., Skipjack Herring, *Alosa chrysochloris*, American Eel, *Anguilla rostrata*) [29]. Among the species that made up at least 1% of the catch, only Silver Carp (*Hypophthalmichthys molitrix*) would seem to fit into that category.

Another possible contribution to a temporal trend is the recent introduction of invasive species to the southern Mississippi River. Silver Carp were introduced decades ago in the southern Mississippi River [30] and over the past 10–15 years have appeared with increasing frequency in Pool 26. Silver Carp is one of the species that appears in our ≥1% species list, but it has never been observed in the dataset outside of Pool 26, so it contributes to differences in the pools with the greatest distances between them (Pool 4 and Pool 26). Silver Carp have probably not reached the northern extent of their range so their contribution to the distance similarity relationship is likely to continue changing over time [15].

### Is temperature an important environment gradient causing the distance-similarity relationship?

One hypothesized mechanism by which distance causes differences in community composition is that distance is a proxy for changing environmental conditions. The Mississippi River is oriented generally north to south, so longer distances correspond to larger latitudinal differences and corresponding changes in thermal regime. In this dataset, models using differences in degree days (a proxy for differences in thermal regime) were a modest improvement over models using distance in terms of fit and effect size, suggesting temperature differences may be responsible for much of the effect of distance on community similarity. This is consistent with the critical role of the thermal environment in determining growth rates and survival of individual fish species, as well as the timing of their spawning events [7,8,31]. Furthermore, a few species seem incapable of reaching high abundances in the southern pools because of temperature constraints (e.g., Northern Pike, Walleye, Yellow Perch [29]).

## Conclusions

The distance-decay function is a fundamental property of how community composition varies across the landscape, but the degree to which it varies over time has been rarely explored. Long-term monitoring of the fish community in the upper Mississippi River offers an interesting opportunity to examine over many years how distance structures fish community composition. In this dataset, we found that for common species the distance-decay function varied over time and may have increased in magnitude. The Mississippi River is aligned mostly north to south, so large distances are tightly correlated to differences in temperature, and temperature differences may account for most of the distance effect in the fish community. If temperature is playing an important role in maintaining these differences, then regional and global changes in climate may alter not just the composition of individual fish communities, but also the distinctiveness of fish communities across the landscape.

## Supporting information

S1 File Statistical appendix. This file includes a description of the R code used to perform the analyses conducted in the methods, including example code. The described code 1) uses publicly available fish composition data to calculate Bray-Curtis similarity indices among Mississippi River pools for each year, 2) takes those Bray-Curtis similarities and models the relationship between similarity and physical distance and 3) takes those Bray-Curtis similarities and models the relationship between similarity, physical distance and degree days (from other publicly available data).
(DOCX)

S2 File Data appendix. This file contains intermediate statistical outputs, such as Bray-Curtis similarity index values and degree days, that are used to build statistical models relating physical distance to Bray-Curtis similarity. While the generating process for these data is included in S1 File, this intermediate data file is included for both transparency and ease of reproduction.
(XLSX)

S1 Table Species observed in the long term resource monitoring program’s daytime electrofishing data. * denotes species that made up more than 1% of the catch in at least 1 pool.
(DOCX)
