# Supplementary material for: Estimating the degree to which distance and temperature differences drive changes in fish community composition over time in the upper Mississippi River
Source: PLoS One. 2019 Dec 2;14(12):e0225630. doi: 10.1371/journal.pone.0225630 (PMC6886860; doi:10.1371/journal.pone.0225630)
Supplement: S1 File — This file includes a description of the R code used to perform the analyses conducted in the methods, including example code. The described code 1) uses publicly available fish composition data to calculate Bray-Curtis similarity indices among Mississippi River pools for each year, 2) takes those Bray-Curtis similarities and models the relationship between similarity and physical distance and 3) takes those Bray-Curtis similarities and models the relationship between similarity, physical distance and degree days (from other publicly available data). (DOCX) [file pone.0225630.s001.docx]

S1 File. Statistical Appendix.

Example code is provided below for statistical analyses performed in this manuscript. This code was written for R version 3.3.2 (R Development Core Team 2014). In this code, everything following a # is part of the description and not part of the actual code. File location data has been simplified, and will have to be inserted for anyone to re-create the analysis.

The example code here has 3 parts:

1. Calculating dissimilarity among pool combinations for each year
2. Modeling the relationship between physical distance (in river kilometers) and Bray-Curtis similarity
3. Modeling the relationship between physical distance (in river kilometers) and Bray-Curtis similarity, with degree days
4. **Calculating dissimilarity among pool combinations for each year**. This code includes a re-sampling protocol to assess the relative influence of relatively small portions of the dataset on the calculated similarities. In this code, similarities are calculated using all species which occurred at least once in the dataset. This was repeated for species that made up at least 1% of the catch in at least 1 pool (see list in S1 Table).

library("plyr")

library("coda", lib.loc="C:/Program Files/R/R-3.1.0/library")

library(rjags)

library(doBy)

#Required packages

LTRMP_FISH_DATA_0507135649 <- read.csv("~//ltrm_fish_data.csv")

#This is the raw data from the LTRMP website.

LTRMP_FISH_DATA_0507135649$sdate<-as.Date(as.character(LTRMP_FISH_DATA_0507135649$sdate),"%m/%d/%Y")

#Putting the date information into the R format

LTRMP_FISH_DATA_0507135649$year<-format(LTRMP_FISH_DATA_0507135649$sdate,"%Y")

#And creating a column for just the year, which is what we are using.

AllDataLTRMP<-LTRMP_FISH_DATA_0507135649[,c(2,3,5,86,10,12,13,15,16,19,73,74,77)]

#There’s a lot of data here, we’re just going to use these columns

LTRMPall6<-subset(AllDataLTRMP,gear=="D")

#Getting just the daytime electrofishing data

#Renamed this file as LTRMPall6 because it fits the code I had written for

# another manuscript

LTRMPall6$catch<-as.numeric(as.character(LTRMPall6$catch))

LTRMPall6$UnID<-paste(LTRMPall6$barcode,LTRMPall6$pool,sep='-')

#Have to create unique ids because some of the barcodes are duplicated

#Actually this was only necessary for the other manuscript, LTRMP data has no

#duplications

fishabundancebypoolandspecies<-ddply(LTRMPall6,.(fishcode,pool),plyr::summarize,TotalCatch=sum(catch,na.rm = TRUE))

fishabundancebypool<-ddply(LTRMPall6,.(pool),plyr::summarize,TotalCatch=sum(catch,na.rm = TRUE))

#This is just to get a sense of how many species occur in the dataset

#This was also used to determine which species to include in the 1%> catch

#species list.

#Removing hybrids, no fish (which is a 'fish species' in this dataset) and a #couple of undefined species. All of these are very rare anyway.

FishNames<-as.character(unique(fishabundancebypoolandspecies[,1]))

FishNames<-FishNames[-c(1,138:145)]

FishNames<-FishNames[FishNames!="BGLE"]

FishNames<-FishNames[FishNames!="BGOS"]

FishNames<-FishNames[FishNames!="BGRS"]

FishNames<-FishNames[FishNames!="BGWM"]

FishNames<-FishNames[FishNames!="CCGF"]

FishNames<-FishNames[FishNames!="GSBG"]

FishNames<-FishNames[FishNames!="GSOS"]

FishNames<-FishNames[FishNames!="GSPS"]

FishNames<-FishNames[FishNames!="GSRS"]

FishNames<-FishNames[FishNames!="GSWM"]

FishNames<-FishNames[FishNames!="LNST"]

FishNames<-FishNames[FishNames!="HQDR"]

FishNames<-FishNames[FishNames!="NFSH"]

FishNames<-FishNames[FishNames!="OSLE"]

FishNames<-FishNames[FishNames!="PSBG"]

FishNames<-FishNames[FishNames!="PSOS"]

FishNames<-FishNames[FishNames!="PSWM"]

FishNames<-FishNames[FishNames!="SBWB"]

FishNames<-FishNames[FishNames!="SCBC"]

FishNames<-FishNames[FishNames!="SGWE"]

FishNames<-FishNames[FishNames!="WPYB"]

FishNames<-FishNames[FishNames!="YOYF"]

UnIDs<- data.frame(UnID=levels(as.factor(LTRMPall6$UnID))

)

#So this loop just re-organizes the data so that it fits into what

#the ‘vegan’ package uses

#I’m aware that there are probably simpler ways to do this.

for(i in 1:length(FishNames))

{

FishCarry<-ddply(subset(LTRMPall6,fishcode==FishNames[i]),.(UnID),plyr::summarize,TotalCatch=sum(catch,na.rm = TRUE))

colnames(FishCarry)<-c("UnID",FishNames[i])

UnIDs<-merge(UnIDs,FishCarry,by="UnID",all.x=TRUE)

}

UnIDs[is.na(UnIDs)] <- 0

#All the NAs here should be zero

colnames(LTRMPall6)

SiteData<-LTRMPall6[,c(14,1,3,4,5,10)]

#remove duplicates

TestSiteData <- unique( SiteData[ ,c(1:6) ] )

ReorganizedData<-merge(UnIDs,TestSiteData)

#I'm going to standardize by minutes of electroshocking here:

colnames(ReorganizedData)

ReorganizedData$effmin<-as.numeric(as.character(ReorganizedData$effmin))

ReorganizedData[,c(2:130)]<- ReorganizedData[,c(2:130)]/ReorganizedData[,135][row(ReorganizedData[,c(2:130)])]

ReorganizedData$StdEffort<-15 #Bringing up to a standard 15 minute run

ReorganizedData[,c(2:130)]<- ReorganizedData[,c(2:130)]*ReorganizedData[,136][row(ReorganizedData[,c(2:130)])]

ReorganizedData<-subset(ReorganizedData,BHMW>-10)

#Getting rid of occasions when effort was not recorded (~70 cases)

#Getting ecological distances

#Getting ecological distances

#Getting ecological distances

#Getting ecological distances

#Getting ecological distances

#Getting ecological distances

#Getting ecological distances

library(ecodist)

library(vegan)

#Fish names for the internal loop

FishNames2<-c("blank",FishNames)

#Creating the data frame outside of the loop

BootstrappedData<-data.frame(Site1=as.character(),Site2=as.character(),

Dissimilarity=as.numeric(),Rep=as.numeric())

#So in this loop we are removing 10% of the data, calculating the Bray-Curtis #similarity among pools, and repeating that 500 times.

for(j in 1:500)

{

BWCSOnly<-subset(ReorganizedData,stratum=="MCB-U")

BWCSOnly<-BWCSOnly[BWCSOnly$pool!="LG",]

BWCSOnly<-BWCSOnly[BWCSOnly$pool!="OR",]

BWCSOnly<-BWCSOnly[BWCSOnly$pool!="",]

MeanBWCSCatch <- unique( BWCSOnly[ ,c(133,134,132) ] )

#Removing 10% of the data

TotalRuns<-length(BWCSOnly$UnID)

Subsetted<-BWCSOnly[sample(1:TotalRuns,TotalRuns*0.9,replace=F),]

for(i in 2:length(FishNames2))

{

Data<-Subsetted[,c(133,134,132,i)]

colnames(Data)<-c("year","pool","stratum","Fish")

FishCarry<-ddply(Data,.(year,stratum,pool),plyr::summarize,TotalCatch=mean(Fish,na.rm = TRUE))

colnames(FishCarry)<-c("year","stratum","pool",FishNames2[i])

MeanBWCSCatch<-merge(MeanBWCSCatch,FishCarry,by=c("year","stratum","pool"),all.x=TRUE)

}

MeanBWCSCatch$year<-substr(MeanBWCSCatch$year,(nchar(MeanBWCSCatch$year)+1)-2,nchar(MeanBWCSCatch$year))

labels<-with(MeanBWCSCatch, paste(pool, year))

rownames(MeanBWCSCatch)<-labels

Bwhabitats<-as.matrix(distance(wisconsin(sqrt(MeanBWCSCatch[,4:25])),"bray-curtis"))

lt<-lower.tri(Bwhabitats)

Distances<-data.frame(Site1 = rownames(Bwhabitats)[row(Bwhabitats)[lt]],

Site2 = rownames(Bwhabitats)[col(Bwhabitats)[lt]],

Dissimilarity =(Bwhabitats)[lt])

Distances$Rep<-j

Distances$Site1<-as.character(Distances$Site1)

Distances$Site2<-as.character(Distances$Site2)

Distances$year1<-as.numeric(substr(Distances$Site1,(nchar(Distances$Site1)+1)-2,nchar(Distances$Site1)))

Distances$year2<-as.numeric(substr(Distances$Site2,(nchar(Distances$Site2)+1)-2,nchar(Distances$Site2)))

Distances$difference<-Distances$year1-Distances$year2

Distances<-Distances[Distances$difference==0,]

#we only want distances within a given year

Distances$year1<-NULL

Distances$year2<-NULL

Distances$difference<-NULL

BootstrappedData<-rbind(BootstrappedData,Distances)

}

#Now we are using those 500 repetitions to calculate an average and standard #deviation for each pool combination

BWSAverages<-ddply(BootstrappedData,.(Site1,Site2),plyr::summarize,DistanceAv=mean(Dissimilarity,na.rm = TRUE))

BWSSD<-ddply(BootstrappedData,.(Site1,Site2),plyr::summarize,Distancesd=sd(Dissimilarity,na.rm = TRUE))

BWSAverages<-merge(BWSAverages,BWSSD ,by=c("Site1","Site2"))

#Saving the data. I then manually added the physical distances to this file #(in river km)

write.csv(BWSAverages,"~/ MCUDistancesAllSpp.csv")

1. **Modeling the relationship between physical distance (in river kilometers) and Bray-Curtis similarity.** In this code, we import data generated in A), that has been combined with the physical distance data and model their relationship. This example uses the ‘all species’ similarities, but the code is virtually identical if the ‘>1% species’ data is used.

library(plyr)

library("lme4", lib.loc="C:/Program Files/R/R-3.2.1/library")

library("AICcmodavg", lib.loc="C:/Program Files/R/R-3.2.1/library")

library("piecewiseSEM")

#Required packages

MCDistDis <- read.csv("~/MCUDistDisAllSpp.csv")

#This file should be in the S2 File (Data Appendix).

MCDistDis$Combination<-paste(as.character(MCDistDis$Site1),as.character(MCDistDis$Site2))

MCDistDis$Combination<-as.factor(MCDistDis$Combination)

MCDistDis$DistanceAv<-1-MCDistDis$DistanceAv

#The Bray-Curtis metric is actually ‘dissimilarity’, so this just switches it #to similarity, basically so it is easier to write about.

#Just calculating some basic descriptive statistics here.

meanData<-aggregate(MCDistDis[, c(1,7:12)], list(MCDistDis$Combination), mean)

meanData$Parameter<-"Mean"

sdData<-aggregate(MCDistDis[, c(1,7:12)], list(MCDistDis$Combination), sd)

sdData$Parameter<-"sd"

maxData<-aggregate(MCDistDis[, c(1,7:12)], list(MCDistDis$Combination), min)

maxData$Parameter<-"max"

minData<-aggregate(MCDistDis[, c(1,7:12)], list(MCDistDis$Combination), max)

minData$Parameter<-"min"

SummaryStats<-rbind(meanData,sdData,maxData,minData)

scale(log(MCDistDis$DistanceAv))

mean(log(MCDistDis$DistanceAv))

sd(log(MCDistDis$DistanceAv))

#This is the parameterization of the multi-level model, including a year #effect

#”Distance” here is Bray-Curtis similarity

Multi.Dis.Dist<-lmer(as.numeric(scale(log(DistanceAv)))~as.numeric(scale(log(Distance)))+(as.numeric(scale(log(Distance)))|Year),

data=MCDistDis,REML = FALSE)

summary(Multi.Dis.Dist)

AICc(Multi.Dis.Dist)

fixef(Multi.Dis.Dist)

confint(Multi.Dis.Dist)

Slopes<-data.frame(coef(Multi.Dis.Dist)$Year)

max(Slopes$as.numeric.scale.log.Distance...)

min(Slopes$as.numeric.scale.log.Distance...)

#extracting the standardized coefficient

rsquared(Multi.Dis.Dist)

#Getting the r squared values for the mixed effect model

#Here’s the null model to compare to the model above

Multi.null<-lmer(as.numeric(scale(log(DistanceAv)))~1+1|Year,

data=MCDistDis,REML = FALSE)

summary(Multi.null)

AICc(Multi.null)

#Looking at the linear trend over time

#Simple regression relating the slope from the 1^st^ model to time (in order)

Time.trend.mod<-lm(as.numeric(scale(Slopes$as.numeric.scale.log.Distance...))~as.numeric(scale(as.numeric(rownames(Slopes)))))

summary(time.trend.mod)

#And comparing that simple linear regression to a null model via AICc

AICc(time.trend.mod)

AICc(lm(as.numeric(scale(Slopes$as.numeric.scale.log.Distance...))~1))

1. **Modeling the relationship between physical distance (in river kilometers) and Bray-Curtis similarity, with degree days.** In this code, we import data generated in A), that has been combined with the physical distance data and differences in degree days and model their relationship. Some data is removed in this process because temperature data in many years is incomplete. This example uses the ‘>1% species’ data.

library(plyr)

library("lme4")

library("AICcmodavg")

library("piecewiseSEM")

MCDistDis <- read.csv("~MCUDistDis.csv")

#This file should be in the S2 File (Data Appendix).

MCDistDis$DistanceAv<-1-MCDistDis$DistanceAv

MCDistDis$Combination<-paste(as.character(MCDistDis$Site1),as.character(MCDistDis$Site2))

MCDistDis$Combination<-as.factor(MCDistDis$Combination)

#Compiling Pool-Pool degree day differences, with 1 or 2 year lag periods

#Pools 26 and 13

levels(MCDistDis$Combination)

MCDistDis26and13<-subset(MCDistDis,Combination=="26 13")

MCDistDis26and13$OneyearagoDD0Diff<-as.numeric(c("NA",MCDistDis26and13$DD0Diff[1:21]))

MCDistDis26and13$TwoyearagoDD0Diff<-as.numeric(c("NA","NA",MCDistDis26and13$DD0Diff[1:20]))

MCDistDis26and13$OneyearagoDD5Diff<-as.numeric(c("NA",MCDistDis26and13$DD5Diff[1:21]))

MCDistDis26and13$TwoyearagoDD5Diff<-as.numeric(c("NA","NA",MCDistDis26and13$DD5Diff[1:20]))

MCDistDis26and13$OneyearagoDD10Diff<-as.numeric(c("NA",MCDistDis26and13$DD10Diff[1:21]))

MCDistDis26and13$TwoyearagoDD10Diff<-as.numeric(c("NA","NA",MCDistDis26and13$DD10Diff[1:20]))

MCDistDis26and13$OneyearagoDD15Diff<-as.numeric(c("NA",MCDistDis26and13$DD15Diff[1:21]))

MCDistDis26and13$TwoyearagoDD15Diff<-as.numeric(c("NA","NA",MCDistDis26and13$DD15Diff[1:20]))

MCDistDis26and13$OneyearagoDD20Diff<-as.numeric(c("NA",MCDistDis26and13$DD20Diff[1:21]))

MCDistDis26and13$TwoyearagoDD20Diff<-as.numeric(c("NA","NA",MCDistDis26and13$DD20Diff[1:20]))

MCDistDis26and13$OneyearagoDD25Diff<-as.numeric(c("NA",MCDistDis26and13$DD25Diff[1:21]))

MCDistDis26and13$TwoyearagoDD25Diff<-as.numeric(c("NA","NA",MCDistDis26and13$DD25Diff[1:20]))

#anything with 2009, 2011 (pool 13), 1998, 2003, 2004, 2009:2014 all (Pool 26) should be tossed

#So, Years 1995:1998,2001,2002,2007,2008 is in for 2-year period

MCDistDis26and132years<-subset(MCDistDis26and13,Year<1999)

MCDistDis26and132years<-subset(MCDistDis26and132years,Year>1994)

MCDistDis26and132years<-rbind(MCDistDis26and132years,subset(MCDistDis26and13,Year==2001))

MCDistDis26and132years<-rbind(MCDistDis26and132years,subset(MCDistDis26and13,Year==2002))

MCDistDis26and132years<-rbind(MCDistDis26and132years,subset(MCDistDis26and13,Year==2007))

MCDistDis26and132years<-rbind(MCDistDis26and132years,subset(MCDistDis26and13,Year==2008))

MCDistDis26and132years$Sum2yearsDD0<-MCDistDis26and132years$OneyearagoDD0Diff+MCDistDis26and132years$TwoyearagoDD0Diff

MCDistDis26and132years$Sum2yearsDD10<-MCDistDis26and132years$OneyearagoDD10Diff+MCDistDis26and132years$TwoyearagoDD10Diff

MCDistDis26and132years$Sum2yearsDD20<-MCDistDis26and132years$OneyearagoDD20Diff+MCDistDis26and132years$TwoyearagoDD20Diff

MCDistDis26and132years$Sum2yearsDD5<-MCDistDis26and132years$OneyearagoDD5Diff+MCDistDis26and132years$TwoyearagoDD5Diff

MCDistDis26and132years$Sum2yearsDD15<-MCDistDis26and132years$OneyearagoDD15Diff+MCDistDis26and132years$TwoyearagoDD15Diff

MCDistDis26and132years$Sum2yearsDD25<-MCDistDis26and132years$OneyearagoDD25Diff+MCDistDis26and132years$TwoyearagoDD25Diff

#anything with 2009, 2011 (pool 13), 1998, 2003, 2004, 2009:2014 all (Pool 26) should be tossed

#So, Years 1994:1998, plus 2001,2002, 2006,2007,2008,2009 is in for 1-year period

MCDistDis26and131years<-subset(MCDistDis26and13,Year>1993)

MCDistDis26and131years<-subset(MCDistDis26and131years,Year<1999)

MCDistDis26and131years<-rbind(MCDistDis26and131years,subset(MCDistDis26and13,Year==2006))

MCDistDis26and131years<-rbind(MCDistDis26and131years,subset(MCDistDis26and13,Year==2007))

MCDistDis26and131years<-rbind(MCDistDis26and131years,subset(MCDistDis26and13,Year==2008))

MCDistDis26and131years<-rbind(MCDistDis26and131years,subset(MCDistDis26and13,Year==2009))

#Pools 13and4

levels(MCDistDis$Combination)

MCDistDis13and4<-subset(MCDistDis,Combination=="13 4")

MCDistDis13and4$OneyearagoDD0Diff<-as.numeric(c("NA",MCDistDis13and4$DD0Diff[1:21]))

MCDistDis13and4$TwoyearagoDD0Diff<-as.numeric(c("NA","NA",MCDistDis13and4$DD0Diff[1:20]))

MCDistDis13and4$OneyearagoDD5Diff<-as.numeric(c("NA",MCDistDis13and4$DD5Diff[1:21]))

MCDistDis13and4$TwoyearagoDD5Diff<-as.numeric(c("NA","NA",MCDistDis13and4$DD5Diff[1:20]))

MCDistDis13and4$OneyearagoDD10Diff<-as.numeric(c("NA",MCDistDis13and4$DD10Diff[1:21]))

MCDistDis13and4$TwoyearagoDD10Diff<-as.numeric(c("NA","NA",MCDistDis13and4$DD10Diff[1:20]))

MCDistDis13and4$OneyearagoDD15Diff<-as.numeric(c("NA",MCDistDis13and4$DD15Diff[1:21]))

MCDistDis13and4$TwoyearagoDD15Diff<-as.numeric(c("NA","NA",MCDistDis13and4$DD15Diff[1:20]))

MCDistDis13and4$OneyearagoDD20Diff<-as.numeric(c("NA",MCDistDis13and4$DD20Diff[1:21]))

MCDistDis13and4$TwoyearagoDD20Diff<-as.numeric(c("NA","NA",MCDistDis13and4$DD20Diff[1:20]))

MCDistDis13and4$OneyearagoDD25Diff<-as.numeric(c("NA",MCDistDis13and4$DD25Diff[1:21]))

MCDistDis13and4$TwoyearagoDD25Diff<-as.numeric(c("NA","NA",MCDistDis13and4$DD25Diff[1:20]))

#anything with 2009, 2011 (pool 13) or 2013, 2014 (pool 4) should be tossed

#So, Years 1995:2009 is in for 2-year period

MCDistDis13and42years<-subset(MCDistDis13and4,Year<2010)

MCDistDis13and42years<-subset(MCDistDis13and42years,Year>1994)

MCDistDis13and42years$Sum2yearsDD0<-MCDistDis13and42years$OneyearagoDD0Diff+MCDistDis13and42years$TwoyearagoDD0Diff

MCDistDis13and42years$Sum2yearsDD10<-MCDistDis13and42years$OneyearagoDD10Diff+MCDistDis13and42years$TwoyearagoDD10Diff

MCDistDis13and42years$Sum2yearsDD20<-MCDistDis13and42years$OneyearagoDD20Diff+MCDistDis13and42years$TwoyearagoDD20Diff

MCDistDis13and42years$Sum2yearsDD5<-MCDistDis13and42years$OneyearagoDD5Diff+MCDistDis13and42years$TwoyearagoDD5Diff

MCDistDis13and42years$Sum2yearsDD15<-MCDistDis13and42years$OneyearagoDD15Diff+MCDistDis13and42years$TwoyearagoDD15Diff

MCDistDis13and42years$Sum2yearsDD25<-MCDistDis13and42years$OneyearagoDD25Diff+MCDistDis13and42years$TwoyearagoDD25Diff

#anything with 2009, 2011 (pool 13) or 2013, 2014 (pool 4) should be tossed

#So, Years 1994:2009, plus 2013 is in for 1-year period

MCDistDis13and41years<-subset(MCDistDis13and4,Year>1993)

MCDistDis13and41years<-subset(MCDistDis13and41years,Year<2010)

MCDistDis13and41years<-rbind(MCDistDis13and41years,subset(MCDistDis13and4,Year==2013))

#Pools 13and8

levels(MCDistDis$Combination)

MCDistDis13and8<-subset(MCDistDis,Combination=="13 8")

MCDistDis13and8$OneyearagoDD0Diff<-as.numeric(c("NA",MCDistDis13and8$DD0Diff[1:21]))

MCDistDis13and8$TwoyearagoDD0Diff<-as.numeric(c("NA","NA",MCDistDis13and8$DD0Diff[1:20]))

MCDistDis13and8$OneyearagoDD5Diff<-as.numeric(c("NA",MCDistDis13and8$DD5Diff[1:21]))

MCDistDis13and8$TwoyearagoDD5Diff<-as.numeric(c("NA","NA",MCDistDis13and8$DD5Diff[1:20]))

MCDistDis13and8$OneyearagoDD10Diff<-as.numeric(c("NA",MCDistDis13and8$DD10Diff[1:21]))

MCDistDis13and8$TwoyearagoDD10Diff<-as.numeric(c("NA","NA",MCDistDis13and8$DD10Diff[1:20]))

MCDistDis13and8$OneyearagoDD15Diff<-as.numeric(c("NA",MCDistDis13and8$DD15Diff[1:21]))

MCDistDis13and8$TwoyearagoDD15Diff<-as.numeric(c("NA","NA",MCDistDis13and8$DD15Diff[1:20]))

MCDistDis13and8$OneyearagoDD20Diff<-as.numeric(c("NA",MCDistDis13and8$DD20Diff[1:21]))

MCDistDis13and8$TwoyearagoDD20Diff<-as.numeric(c("NA","NA",MCDistDis13and8$DD20Diff[1:20]))

MCDistDis13and8$OneyearagoDD25Diff<-as.numeric(c("NA",MCDistDis13and8$DD25Diff[1:21]))

MCDistDis13and8$TwoyearagoDD25Diff<-as.numeric(c("NA","NA",MCDistDis13and8$DD25Diff[1:20]))

#anything with 2009, 2011 (pool 13) should be tossed

#So, Years 1995:2009, plus 2014 is in for 2-year period

MCDistDis13and82years<-subset(MCDistDis13and8,Year<2010)

MCDistDis13and82years<-subset(MCDistDis13and82years,Year>1994)

MCDistDis13and82years<-rbind(MCDistDis13and82years,subset(MCDistDis13and8,Year==2014))

MCDistDis13and82years$Sum2yearsDD0<-MCDistDis13and82years$OneyearagoDD0Diff+MCDistDis13and82years$TwoyearagoDD0Diff

MCDistDis13and82years$Sum2yearsDD10<-MCDistDis13and82years$OneyearagoDD10Diff+MCDistDis13and82years$TwoyearagoDD10Diff

MCDistDis13and82years$Sum2yearsDD20<-MCDistDis13and82years$OneyearagoDD20Diff+MCDistDis13and82years$TwoyearagoDD20Diff

MCDistDis13and82years$Sum2yearsDD5<-MCDistDis13and82years$OneyearagoDD5Diff+MCDistDis13and82years$TwoyearagoDD5Diff

MCDistDis13and82years$Sum2yearsDD15<-MCDistDis13and82years$OneyearagoDD15Diff+MCDistDis13and82years$TwoyearagoDD15Diff

MCDistDis13and82years$Sum2yearsDD25<-MCDistDis13and82years$OneyearagoDD25Diff+MCDistDis13and82years$TwoyearagoDD25Diff

#anything with 2009, 2011 (pool 13) should be tossed

#So, Years 1994:2009, plus 2011, 2013:2014 is in for 1-year period

MCDistDis13and81years<-subset(MCDistDis13and8,Year>1993)

MCDistDis13and81years<-subset(MCDistDis13and81years,Year<2010)

MCDistDis13and81years<-rbind(MCDistDis13and81years,subset(MCDistDis13and8,Year==2011))

MCDistDis13and81years<-rbind(MCDistDis13and81years,subset(MCDistDis13and8,Year==2013))

MCDistDis13and81years<-rbind(MCDistDis13and81years,subset(MCDistDis13and8,Year==2014))

#Pools 26and4

#Pools 26and4

#Pools 26and4

#Pools 26and4

levels(MCDistDis$Combination)

MCDistDis26and4<-subset(MCDistDis,Combination=="26 4")

MCDistDis26and4$OneyearagoDD0Diff<-as.numeric(c("NA",MCDistDis26and4$DD0Diff[1:21]))

MCDistDis26and4$TwoyearagoDD0Diff<-as.numeric(c("NA","NA",MCDistDis26and4$DD0Diff[1:20]))

MCDistDis26and4$OneyearagoDD5Diff<-as.numeric(c("NA",MCDistDis26and4$DD5Diff[1:21]))

MCDistDis26and4$TwoyearagoDD5Diff<-as.numeric(c("NA","NA",MCDistDis26and4$DD5Diff[1:20]))

MCDistDis26and4$OneyearagoDD10Diff<-as.numeric(c("NA",MCDistDis26and4$DD10Diff[1:21]))

MCDistDis26and4$TwoyearagoDD10Diff<-as.numeric(c("NA","NA",MCDistDis26and4$DD10Diff[1:20]))

MCDistDis26and4$OneyearagoDD15Diff<-as.numeric(c("NA",MCDistDis26and4$DD15Diff[1:21]))

MCDistDis26and4$TwoyearagoDD15Diff<-as.numeric(c("NA","NA",MCDistDis26and4$DD15Diff[1:20]))

MCDistDis26and4$OneyearagoDD20Diff<-as.numeric(c("NA",MCDistDis26and4$DD20Diff[1:21]))

MCDistDis26and4$TwoyearagoDD20Diff<-as.numeric(c("NA","NA",MCDistDis26and4$DD20Diff[1:20]))

MCDistDis26and4$OneyearagoDD25Diff<-as.numeric(c("NA",MCDistDis26and4$DD25Diff[1:21]))

MCDistDis26and4$TwoyearagoDD25Diff<-as.numeric(c("NA","NA",MCDistDis26and4$DD25Diff[1:20]))

#anything with 2013 2014 (pool 4) & 1998, 03:04, 09:14 (pool 26) should be tossed

#So, Years 1995:1997, plus 2001,2002,2007,2008 is in for 2-year period

MCDistDis26and42years<-subset(MCDistDis26and4,Year<1998)

MCDistDis26and42years<-subset(MCDistDis26and42years,Year>1994)

MCDistDis26and42years<-rbind(MCDistDis26and42years,subset(MCDistDis26and4,Year==2001))

MCDistDis26and42years<-rbind(MCDistDis26and42years,subset(MCDistDis26and4,Year==2002))

MCDistDis26and42years<-rbind(MCDistDis26and42years,subset(MCDistDis26and4,Year==2007))

MCDistDis26and42years<-rbind(MCDistDis26and42years,subset(MCDistDis26and4,Year==2008))

MCDistDis26and42years$Sum2yearsDD0<-MCDistDis26and42years$OneyearagoDD0Diff+MCDistDis26and42years$TwoyearagoDD0Diff

MCDistDis26and42years$Sum2yearsDD10<-MCDistDis26and42years$OneyearagoDD10Diff+MCDistDis26and42years$TwoyearagoDD10Diff

MCDistDis26and42years$Sum2yearsDD20<-MCDistDis26and42years$OneyearagoDD20Diff+MCDistDis26and42years$TwoyearagoDD20Diff

MCDistDis26and42years$Sum2yearsDD5<-MCDistDis26and42years$OneyearagoDD5Diff+MCDistDis26and42years$TwoyearagoDD5Diff

MCDistDis26and42years$Sum2yearsDD15<-MCDistDis26and42years$OneyearagoDD15Diff+MCDistDis26and42years$TwoyearagoDD15Diff

MCDistDis26and42years$Sum2yearsDD25<-MCDistDis26and42years$OneyearagoDD25Diff+MCDistDis26and42years$TwoyearagoDD25Diff

#anything with 2013 2014 (pool 4) & 1998, 03:04, 09:14 (pool 26) should be tossed

#So, Years 1994:1998, plus 2000-2003, 2006-2009 is in for 1-year period

MCDistDis26and41years<-subset(MCDistDis26and4,Year>1993)

MCDistDis26and41years<-subset(MCDistDis26and41years,Year<1999)

MCDistDis26and41years<-rbind(MCDistDis26and41years,subset(MCDistDis26and4,Year==2000))

MCDistDis26and41years<-rbind(MCDistDis26and41years,subset(MCDistDis26and4,Year==2001))

MCDistDis26and41years<-rbind(MCDistDis26and41years,subset(MCDistDis26and4,Year==2002))

MCDistDis26and41years<-rbind(MCDistDis26and41years,subset(MCDistDis26and4,Year==2003))

MCDistDis26and41years<-rbind(MCDistDis26and41years,subset(MCDistDis26and4,Year==2006))

MCDistDis26and41years<-rbind(MCDistDis26and41years,subset(MCDistDis26and4,Year==2007))

MCDistDis26and41years<-rbind(MCDistDis26and41years,subset(MCDistDis26and4,Year==2008))

MCDistDis26and41years<-rbind(MCDistDis26and41years,subset(MCDistDis26and4,Year==2009))

#Pools 26and8

#Pools 26and8

#Pools 26and8

#Pools 26and8

levels(MCDistDis$Combination)

MCDistDis26and8<-subset(MCDistDis,Combination=="26 8")

MCDistDis26and8$OneyearagoDD0Diff<-as.numeric(c("NA",MCDistDis26and8$DD0Diff[1:21]))

MCDistDis26and8$TwoyearagoDD0Diff<-as.numeric(c("NA","NA",MCDistDis26and8$DD0Diff[1:20]))

MCDistDis26and8$OneyearagoDD5Diff<-as.numeric(c("NA",MCDistDis26and8$DD5Diff[1:21]))

MCDistDis26and8$TwoyearagoDD5Diff<-as.numeric(c("NA","NA",MCDistDis26and8$DD5Diff[1:20]))

MCDistDis26and8$OneyearagoDD10Diff<-as.numeric(c("NA",MCDistDis26and8$DD10Diff[1:21]))

MCDistDis26and8$TwoyearagoDD10Diff<-as.numeric(c("NA","NA",MCDistDis26and8$DD10Diff[1:20]))

MCDistDis26and8$OneyearagoDD15Diff<-as.numeric(c("NA",MCDistDis26and8$DD15Diff[1:21]))

MCDistDis26and8$TwoyearagoDD15Diff<-as.numeric(c("NA","NA",MCDistDis26and8$DD15Diff[1:20]))

MCDistDis26and8$OneyearagoDD20Diff<-as.numeric(c("NA",MCDistDis26and8$DD20Diff[1:21]))

MCDistDis26and8$TwoyearagoDD20Diff<-as.numeric(c("NA","NA",MCDistDis26and8$DD20Diff[1:20]))

MCDistDis26and8$OneyearagoDD25Diff<-as.numeric(c("NA",MCDistDis26and8$DD25Diff[1:21]))

MCDistDis26and8$TwoyearagoDD25Diff<-as.numeric(c("NA","NA",MCDistDis26and8$DD25Diff[1:20]))

#anything with 1998, 03:04, 09:14 (pool 26) should be tossed

#So, Years 1995:1997, plus 2001,2002,2007,2008 is in for 2-year period

MCDistDis26and82years<-subset(MCDistDis26and8,Year<1998)

MCDistDis26and82years<-subset(MCDistDis26and82years,Year>1994)

MCDistDis26and82years<-rbind(MCDistDis26and82years,subset(MCDistDis26and8,Year==2001))

MCDistDis26and82years<-rbind(MCDistDis26and82years,subset(MCDistDis26and8,Year==2002))

MCDistDis26and82years<-rbind(MCDistDis26and82years,subset(MCDistDis26and8,Year==2007))

MCDistDis26and82years<-rbind(MCDistDis26and82years,subset(MCDistDis26and8,Year==2008))

MCDistDis26and82years$Sum2yearsDD0<-MCDistDis26and82years$OneyearagoDD0Diff+MCDistDis26and82years$TwoyearagoDD0Diff

MCDistDis26and82years$Sum2yearsDD10<-MCDistDis26and82years$OneyearagoDD10Diff+MCDistDis26and82years$TwoyearagoDD10Diff

MCDistDis26and82years$Sum2yearsDD20<-MCDistDis26and82years$OneyearagoDD20Diff+MCDistDis26and82years$TwoyearagoDD20Diff

MCDistDis26and82years$Sum2yearsDD5<-MCDistDis26and82years$OneyearagoDD5Diff+MCDistDis26and82years$TwoyearagoDD5Diff

MCDistDis26and82years$Sum2yearsDD15<-MCDistDis26and82years$OneyearagoDD15Diff+MCDistDis26and82years$TwoyearagoDD15Diff

MCDistDis26and82years$Sum2yearsDD25<-MCDistDis26and82years$OneyearagoDD25Diff+MCDistDis26and82years$TwoyearagoDD25Diff

#anything with 1998, 03:04, 09:14 (pool 26) should be tossed

#So, Years 1994:1998, plus 2000-2003, 2006-2009 is in for 1-year period

MCDistDis26and81years<-subset(MCDistDis26and8,Year>1993)

MCDistDis26and81years<-subset(MCDistDis26and81years,Year<1999)

MCDistDis26and81years<-rbind(MCDistDis26and81years,subset(MCDistDis26and8,Year==2000))

MCDistDis26and81years<-rbind(MCDistDis26and81years,subset(MCDistDis26and8,Year==2001))

MCDistDis26and81years<-rbind(MCDistDis26and81years,subset(MCDistDis26and8,Year==2002))

MCDistDis26and81years<-rbind(MCDistDis26and81years,subset(MCDistDis26and8,Year==2003))

MCDistDis26and81years<-rbind(MCDistDis26and81years,subset(MCDistDis26and8,Year==2006))

MCDistDis26and81years<-rbind(MCDistDis26and81years,subset(MCDistDis26and8,Year==2007))

MCDistDis26and81years<-rbind(MCDistDis26and81years,subset(MCDistDis26and8,Year==2008))

MCDistDis26and81years<-rbind(MCDistDis26and81years,subset(MCDistDis26and8,Year==2009))

#Pools 8and4

#Pools 8and4

#Pools 8and4

#Pools 8and4

levels(MCDistDis$Combination)

MCDistDis8and4<-subset(MCDistDis,Combination=="8 4")

MCDistDis8and4$OneyearagoDD0Diff<-as.numeric(c("NA",MCDistDis8and4$DD0Diff[1:21]))

MCDistDis8and4$TwoyearagoDD0Diff<-as.numeric(c("NA","NA",MCDistDis8and4$DD0Diff[1:20]))

MCDistDis8and4$OneyearagoDD5Diff<-as.numeric(c("NA",MCDistDis8and4$DD5Diff[1:21]))

MCDistDis8and4$TwoyearagoDD5Diff<-as.numeric(c("NA","NA",MCDistDis8and4$DD5Diff[1:20]))

MCDistDis8and4$OneyearagoDD10Diff<-as.numeric(c("NA",MCDistDis8and4$DD10Diff[1:21]))

MCDistDis8and4$TwoyearagoDD10Diff<-as.numeric(c("NA","NA",MCDistDis8and4$DD10Diff[1:20]))

MCDistDis8and4$OneyearagoDD15Diff<-as.numeric(c("NA",MCDistDis8and4$DD15Diff[1:21]))

MCDistDis8and4$TwoyearagoDD15Diff<-as.numeric(c("NA","NA",MCDistDis8and4$DD15Diff[1:20]))

MCDistDis8and4$OneyearagoDD20Diff<-as.numeric(c("NA",MCDistDis8and4$DD20Diff[1:21]))

MCDistDis8and4$TwoyearagoDD20Diff<-as.numeric(c("NA","NA",MCDistDis8and4$DD20Diff[1:20]))

MCDistDis8and4$OneyearagoDD25Diff<-as.numeric(c("NA",MCDistDis8and4$DD25Diff[1:21]))

MCDistDis8and4$TwoyearagoDD25Diff<-as.numeric(c("NA","NA",MCDistDis8and4$DD25Diff[1:20]))

#anything with 2013 or 2014 (pool 4) should be tossed

#So, Years 1995:2013, is in for 2-year period

MCDistDis8and42years<-subset(MCDistDis8and4,Year<2014)

MCDistDis8and42years<-subset(MCDistDis8and42years,Year>1994)

MCDistDis8and42years$Sum2yearsDD0<-MCDistDis8and42years$OneyearagoDD0Diff+MCDistDis8and42years$TwoyearagoDD0Diff

MCDistDis8and42years$Sum2yearsDD10<-MCDistDis8and42years$OneyearagoDD10Diff+MCDistDis8and42years$TwoyearagoDD10Diff

MCDistDis8and42years$Sum2yearsDD20<-MCDistDis8and42years$OneyearagoDD20Diff+MCDistDis8and42years$TwoyearagoDD20Diff

MCDistDis8and42years$Sum2yearsDD5<-MCDistDis8and42years$OneyearagoDD5Diff+MCDistDis8and42years$TwoyearagoDD5Diff

MCDistDis8and42years$Sum2yearsDD15<-MCDistDis8and42years$OneyearagoDD15Diff+MCDistDis8and42years$TwoyearagoDD15Diff

MCDistDis8and42years$Sum2yearsDD25<-MCDistDis8and42years$OneyearagoDD25Diff+MCDistDis8and42years$TwoyearagoDD25Diff

#anything with 2013 or 2014 (pool 4) should be tossed

#So, Years 1994:2013is in for 1-year period

MCDistDis8and41years<-subset(MCDistDis8and4,Year>1993)

MCDistDis8and41years<-subset(MCDistDis8and41years,Year<2014)

AllData2years<-rbind(MCDistDis8and42years,MCDistDis26and82years,MCDistDis26and132years,MCDistDis26and42years,MCDistDis13and82years,MCDistDis13and42years)

cor(AllData2years$OneyearagoDD0Diff,AllData2years$Distance)

cor(AllData2years$OneyearagoDD20Diff,AllData2years$Distance)

cor(AllData2years$Sum2yearsDD0,AllData2years$Distance)

cor(AllData2years$Sum2yearsDD20,AllData2years$Distance)

#Model selection- All models listed

#Model without DD

Multi.Dis.Dist<-lmer(as.numeric(scale(log(DistanceAv)))~as.numeric(scale(log(Distance)))+(as.numeric(scale(log(Distance)))|Year),

data=AllData2years,REML = FALSE)

#Model with DD0 1 previous year

Multi.Dis.Dist.DD01<-lmer(as.numeric(scale(log(DistanceAv)))~as.numeric(scale(log(Distance)))+as.numeric(scale(OneyearagoDD0Diff))+(as.numeric(scale(log(Distance)))|Year),

data=AllData2years,REML = FALSE)

#Model with DD0 2 previous years

Multi.Dis.Dist.DD02<-lmer(as.numeric(scale(log(DistanceAv)))~as.numeric(scale(log(Distance)))+as.numeric(scale(Sum2yearsDD0))+(as.numeric(scale(log(Distance)))|Year),

data=AllData2years,REML = FALSE)

#Model with DD15 1 previous year

Multi.Dis.Dist.DD151<-lmer(as.numeric(scale(log(DistanceAv)))~as.numeric(scale(log(Distance)))+as.numeric(scale(OneyearagoDD15Diff))+(as.numeric(scale(log(Distance)))|Year),

data=AllData2years,REML = FALSE)

#Model with DD15 2 previous years

Multi.Dis.Dist.DD152<-lmer(as.numeric(scale(log(DistanceAv)))~as.numeric(scale(log(Distance)))+as.numeric(scale(Sum2yearsDD15))+(as.numeric(scale(log(Distance)))|Year),

data=AllData2years,REML = FALSE)

#No Dist with DD15 2 previous years

Multi.Dis.DD152<-lmer(as.numeric(scale(log(DistanceAv)))~as.numeric(scale(Sum2yearsDD15))+(1|Year),

data=AllData2years,REML = FALSE)

#No Dist with DD15 1 previous years

Multi.Dis.DD151<-lmer(as.numeric(scale(log(DistanceAv)))~as.numeric(scale(OneyearagoDD15Diff))+(1|Year),

data=AllData2years,REML = FALSE)

#No Dist with DD0 2 previous years

Multi.Dis.DD02<-lmer(as.numeric(scale(log(DistanceAv)))~as.numeric(scale(Sum2yearsDD0))+(1|Year),

data=AllData2years,REML = FALSE)

#No Dist with DD0 1 previous years

Multi.Dis.DD01<-lmer(as.numeric(scale(log(DistanceAv)))~as.numeric(scale(OneyearagoDD0Diff))+(1|Year),

data=AllData2years,REML = FALSE)

#Model with DD0 1 previous year

Multi.Dis.Dist.DD01<-lmer(as.numeric(scale(log(DistanceAv)))~as.numeric(scale(log(Distance)))+as.numeric(scale(OneyearagoDD0Diff))+(as.numeric(scale(log(Distance)))|Year),

data=AllData2years,REML = FALSE)

#Model with DD0 2 previous years

Multi.Dis.Dist.DD02<-lmer(as.numeric(scale(log(DistanceAv)))~as.numeric(scale(log(Distance)))+as.numeric(scale(Sum2yearsDD0))+(as.numeric(scale(log(Distance)))|Year),

data=AllData2years,REML = FALSE)

#No Dist with DD10 2 previous years

Multi.Dis.DD102<-lmer(as.numeric(scale(log(DistanceAv)))~as.numeric(scale(Sum2yearsDD10))+(1|Year),

data=AllData2years,REML = FALSE)

#No Dist with DD10 1 previous years

Multi.Dis.DD101<-lmer(as.numeric(scale(log(DistanceAv)))~as.numeric(scale(OneyearagoDD10Diff))+(1|Year),

data=AllData2years,REML = FALSE)

#Model with DD10 1 previous year

Multi.Dis.Dist.DD101<-lmer(as.numeric(scale(log(DistanceAv)))~as.numeric(scale(log(Distance)))+as.numeric(scale(OneyearagoDD10Diff))+(as.numeric(scale(log(Distance)))|Year),

data=AllData2years,REML = FALSE)

#Model with DD10 2 previous years

Multi.Dis.Dist.DD102<-lmer(as.numeric(scale(log(DistanceAv)))~as.numeric(scale(log(Distance)))+as.numeric(scale(Sum2yearsDD10))+(as.numeric(scale(log(Distance)))|Year),

data=AllData2years,REML = FALSE)

#No Dist with DD20 2 previous years

Multi.Dis.DD202<-lmer(as.numeric(scale(log(DistanceAv)))~as.numeric(scale(Sum2yearsDD20))+(1|Year),

data=AllData2years,REML = FALSE)

#No Dist with DD20 1 previous years

Multi.Dis.DD201<-lmer(as.numeric(scale(log(DistanceAv)))~as.numeric(scale(OneyearagoDD20Diff))+(1|Year),

data=AllData2years,REML = FALSE)

#Model with DD20 1 previous year

Multi.Dis.Dist.DD201<-lmer(as.numeric(scale(log(DistanceAv)))~as.numeric(scale(log(Distance)))+as.numeric(scale(OneyearagoDD20Diff))+(as.numeric(scale(log(Distance)))|Year),

data=AllData2years,REML = FALSE)

#Model with DD20 2 previous years

Multi.Dis.Dist.DD202<-lmer(as.numeric(scale(log(DistanceAv)))~as.numeric(scale(log(Distance)))+as.numeric(scale(Sum2yearsDD20))+(as.numeric(scale(log(Distance)))|Year),

data=AllData2years,REML = FALSE)

#No Dist with DD25 2 previous years

Multi.Dis.DD252<-lmer(as.numeric(scale(log(DistanceAv)))~as.numeric(scale(Sum2yearsDD25))+(1|Year),

data=AllData2years,REML = FALSE)

#No Dist with DD25 1 previous years

Multi.Dis.DD251<-lmer(as.numeric(scale(log(DistanceAv)))~as.numeric(scale(OneyearagoDD25Diff))+(1|Year),

data=AllData2years,REML = FALSE)

#Model with DD25 1 previous year

Multi.Dis.Dist.DD251<-lmer(as.numeric(scale(log(DistanceAv)))~as.numeric(scale(log(Distance)))+as.numeric(scale(OneyearagoDD25Diff))+(as.numeric(scale(log(Distance)))|Year),

data=AllData2years,REML = FALSE)

#Model with DD25 2 previous years

Multi.Dis.Dist.DD252<-lmer(as.numeric(scale(log(DistanceAv)))~as.numeric(scale(log(Distance)))+as.numeric(scale(Sum2yearsDD25))+(as.numeric(scale(log(Distance)))|Year),

data=AllData2years,REML = FALSE)

#No Dist with DD5 2 previous years

Multi.Dis.DD52<-lmer(as.numeric(scale(log(DistanceAv)))~as.numeric(scale(Sum2yearsDD5))+(1|Year),

data=AllData2years,REML = FALSE)

#No Dist with DD5 1 previous years

Multi.Dis.DD51<-lmer(as.numeric(scale(log(DistanceAv)))~as.numeric(scale(OneyearagoDD5Diff))+(1|Year),

data=AllData2years,REML = FALSE)

#Model with DD5 1 previous year

Multi.Dis.Dist.DD51<-lmer(as.numeric(scale(log(DistanceAv)))~as.numeric(scale(log(Distance)))+as.numeric(scale(OneyearagoDD5Diff))+(as.numeric(scale(log(Distance)))|Year),

data=AllData2years,REML = FALSE)

#Model with DD5 2 previous years

Multi.Dis.Dist.DD52<-lmer(as.numeric(scale(log(DistanceAv)))~as.numeric(scale(log(Distance)))+as.numeric(scale(Sum2yearsDD5))+(as.numeric(scale(log(Distance)))|Year),

data=AllData2years,REML = FALSE)

Multi.Null<-lmer(as.numeric(scale(log(DistanceAv)))~1+(1|Year),

data=AllData2years,REML = FALSE)

modelList<-c(

Multi.Dis.Dist,

Multi.Dis.Dist.DD01,

Multi.Dis.Dist.DD02,

Multi.Dis.Dist.DD151,

Multi.Dis.Dist.DD152,

Multi.Dis.DD152,

Multi.Dis.DD151,

Multi.Dis.DD02,

Multi.Dis.DD01,

Multi.Null,

Multi.Dis.DD102,

Multi.Dis.DD101,

Multi.Dis.Dist.DD101,

Multi.Dis.Dist.DD102,

Multi.Dis.DD202,

Multi.Dis.DD201,

Multi.Dis.Dist.DD201,

Multi.Dis.Dist.DD202,

Multi.Dis.DD252,

Multi.Dis.DD251,

Multi.Dis.Dist.DD251,

Multi.Dis.Dist.DD252,

Multi.Dis.DD52,

Multi.Dis.DD51,

Multi.Dis.Dist.DD51,

Multi.Dis.Dist.DD52

)

ModelR2s.1variable<-rsquared(modelList)

ModelR2s.1variable$Model<-c("Multi.Dis.Dist",

"Multi.Dis.Dist.DD01",

"Multi.Dis.Dist.DD02",

"Multi.Dis.Dist.DD151",

"Multi.Dis.Dist.DD152",

"Multi.Dis.DD152",

"Multi.Dis.DD151",

"Multi.Dis.DD02",

"Multi.Dis.DD01",

"Multi.Null",

"Multi.Dis.DD102",

"Multi.Dis.DD101",

"Multi.Dis.Dist.DD101",

"Multi.Dis.Dist.DD102",

"Multi.Dis.DD202",

"Multi.Dis.DD201",

"Multi.Dis.Dist.DD201",

"Multi.Dis.Dist.DD202",

"Multi.Dis.DD252",

"Multi.Dis.DD251",

"Multi.Dis.Dist.DD251",

"Multi.Dis.Dist.DD252",

"Multi.Dis.DD52",

"Multi.Dis.DD51",

"Multi.Dis.Dist.DD51",

"Multi.Dis.Dist.DD52"

)

ModelswAICC<-data.frame(sapply(modelList, AICc))

ModelswAICC$AICc<-ModelswAICC$sapply.modelList..AICc.

ModelswAICC$sapply.modelList..AICc.<-NULL

ModelswAICC$Model<-c("Multi.Dis.Dist",

"Multi.Dis.Dist.DD01",

"Multi.Dis.Dist.DD02",

"Multi.Dis.Dist.DD151",

"Multi.Dis.Dist.DD152",

"Multi.Dis.DD152",

"Multi.Dis.DD151",

"Multi.Dis.DD02",

"Multi.Dis.DD01",

"Multi.Null",

"Multi.Dis.DD102",

"Multi.Dis.DD101",

"Multi.Dis.Dist.DD101",

"Multi.Dis.Dist.DD102",

"Multi.Dis.DD202",

"Multi.Dis.DD201",

"Multi.Dis.Dist.DD201",

"Multi.Dis.Dist.DD202",

"Multi.Dis.DD252",

"Multi.Dis.DD251",

"Multi.Dis.Dist.DD251",

"Multi.Dis.Dist.DD252",

"Multi.Dis.DD52",

"Multi.Dis.DD51",

"Multi.Dis.Dist.DD51",

"Multi.Dis.Dist.DD52"

)

ModelswAICC$dAICc<-ModelswAICC$AICc-min(ModelswAICC$AICc)

RankedAICc<-ModelswAICC[order(ModelswAICC$dAICc),]

class(ModelR2s.1variable$Model)

RankedAICc<-merge(RankedAICc,ModelR2s.1variable, by="Model")

RankedAICc<-RankedAICc[order(RankedAICc$dAICc),]

summary(Multi.Dis.Dist.DD52)

confint(Multi.Dis.Dist.DD52)
