# Supplementary material for: Estimating the degree to which distance and temperature differences drive changes in fish community composition over time in the upper Mississippi River
Source: PLoS One. 2019 Dec 2;14(12):e0225630. doi: 10.1371/journal.pone.0225630 (PMC6886860; doi:10.1371/journal.pone.0225630)
Supplement: S1 Table — * denotes species that made up more than 1% of the catch in at least 1 pool. (DOCX) [file pone.0225630.s003.docx]

S1 Table. Species observed in the Long Term Resource Monitoring Program’s daytime electrofishing data. * denotes species that made up more than 1% of the catch in at least 1 pool.

| Common name | Species Name |
| --- | --- |
| American brook lamprey | *Lethenteron appendix* |
| American eel | *Anguilla rostrate* |
| Banded darter | *Etheostoma zonale* |
| Bigeye shiner | *Notropis boops* |
| Bighead carp | *Hypophthalmichthys nobilis* |
| Bullhead minnow* | *Pimephales vigilax* |
| Black buffalo | *Ictiobus niger* |
| Black bullhead | *Ameiurus melas* |
| Black crappie* | *Pomoxis nigromaculatus* |
| Brook stickleback | *Culaea inconstans* |
| Brook silverside | *Labidesthes sicculus* |
| Blue catfish | *Ictalurus furcatus* |
| Bluegill* | *Lepomis macrochirus* |
| Bigmouth buffalo | *Ictiobus cyprinellus* |
| Brown bullhead | *Ameiurus nebulosus* |
| Bluntnose darter | *Etheostoma chlorosomum* |
| Bluntnose minnow | *Pimephales notatus* |
| Blackspotted topminnow | *Fundulus olivaceus* |
| Burbot | *Lota lota* |
| Blackside darter | *Percina maculata* |
| Brassy minnow | *Hybognathus hankinsoni* |
| Blacktail shiner | *Cyprinella venusta* |
| Blackstripe topminnow | *Fundulus notatus* |
| Blue sucker | *Cycleptus elongatus* |
| Bowfin | *Amia calva* |
| Common carp* | *Cyprinus carpio* |
| Creek chub | *Semotilus atromaculatus* |
| Creek chubsucker | *Erimyzon oblongus* |
| Crystal darter | *Crystallaria asprella* |
| Central stoneroller | *Campostoma anomalum* |
| Central mudminnow | *Umbra limi* |
| Common shiner* | *Luxilus cornutus* |
| Channel catfish* | *Ictalurus punctatus* |
| Chestnut lamprey | *Ichthyomyzon castaneus* |
| Channel shiner | *Notropis wickliffi* |
| Dusky darter | *Percina sciera* |
| Emerald shiner* | *Notropis atherinoides* |
| Flathead catfish | *Pylodictis olivaris* |
| Fathead minnow | *Pimephales promelas* |
| Freckled madtom | *Noturus nocturnus* |
| Fantail darter | *Etheostoma flabellare* |
| Freshwater drum* | *Aplodinotus grunniens* |
| Goldeye | *Hiodon alosoides* |
| Goldfish | *Carassius auratus* |
| Golden redhorse | *Moxostoma erythrurum* |
| Golden shiner* | *Notemigonus crysoleucas* |
| Green sunfish* | *Lepomis cyanellus* |
| Grass carp | *Ctenopharyngodon idella* |
| Grass pickerel | *Esox americanus* |
| Gizzard shad* | *Dorosoma cepedianum* |
| Highfin carpsucker | *Carpiodes velifer* |
| Hornyhead chub | *Nocomis biguttatus* |
| Inland silverside | *Menidia beryllina* |
| Iowa darter | *Etheostoma exile* |
| Johnny darter | *Etheostoma nigrum* |
| Longear sunfish | *Lepomis megalotis* |
| Logperch | *Percina caprodes* |
| Lake sturgeon | *Acipenser fulvescens* |
| Largemouth bass* | *Micropterus salmoides* |
| Longnose gar | *Lepisosteus osseus* |
| Mud darter | *Etheostoma asprigene* |
| Mimic shiner* | *Notropis volucellus* |
| Mooneye | *Hiodon tergisus* |
| Western mosquitofish | *Gambusia affinis* |
| Muskellunge | *Esox masquinongy* |
| Northern hog sucker | *Hypentelium nigricans* |
| Northern pike | *Esox lucius* |
| Northern studfish | *Fundulus catenatus* |
| Orangespotted sunfish* | *Lepomis humilis* |
| Orangethroat darter | *Etheostoma spectabile* |
| Paddlefish | *Polyodon spathula* |
| Pallid shiner | *Hybopsis amnis* |
| Pugnose minnow | *Opsopoeodus emiliae* |
| Plains minnow | *Hybognathus placitus* |
| Pumpkinseed* | *Lepomis gibbosus* |
| Pirate perch | *Aphredoderus sayanus* |
| Quillback | *Carpiodes cyprinus* |
| Red shiner | *Cyprinella lutrensis* |
| Redear sunfish | *Lepomis microlophus* |
| Rock bass | *Ambloplites rupestris* |
| River darter | *Percina shumardi* |
| Redspotted sunfish | *Lepomis miniatus* |
| River carpsucker | *Carpiodes carpio* |
| River redhorse | *Moxostoma carinatum* |
| River shiner* | *Notropis blennius* |
| Silverband shiner | *Notropis shumardi* |
| Striped bass | *Morone saxatilis* |
| Sicklefin chub | *Macrhybopsis meeki* |
| Spotfin shiner* | *Cyprinella spiloptera* |
| Sauger | *Sander canadensis* |
| Slenderhead darter | *Percina phoxocephala* |
| Shorthead redhorse* | *Moxostoma macrolepidotum* |
| Skipjack herring | *Alosa chrysochloris* |
| Speckled chub | *Macrhybopsis aestivalis* |
| Smallmouth buffalo* | *Ictiobus bubalus* |
| Smallmouth bass | *Micropterus dolomieu* |
| Suckermouth minnow | *Phenacobius mirabilis* |
| Shortnose gar | *Lepisosteus platostomus* |
| Shovelnose sturgeon | *Scaphirhynchus platorynchus* |
| Sand shiner | *Notropis stramineus* |
| Striped mullet | *Mugil cephalus* |
| Spotted sucker | *Minytrema melanops* |
| Striped shiner | *Luxilus chrysocephalus* |
| Spotted bass | *Micropterus punctulatus* |
| Stonecat | *Noturus flavus* |
| Spotted gar | *Lepisosteus oculatus* |
| Spottail shiner | *Notropis hudsonius* |
| Silver chub | *Macrhybopsis storeriana* |
| Silver carp* | *Hypophthalmichthys molitrix* |
| Silver lamprey | *Ichthyomyzon unicuspis* |
| Mississippi silvery minnow | *Hybognathus nuchalis* |
| Silver redhorse | *Moxostoma anisurum* |
| Threadfin shad | *Dorosoma petenense* |
| Tadpole madtom | *Noturus gyrinus* |
| Trout perch | *Percopsis omiscomaycus* |
| Weed shiner | *Notropis texanus* |
| Walleye | *Sander vitreus* |
| Warmouth | *Lepomis gulosus* |
| Western sand darter | *Ammocrypta clara* |
| Wedgespot shiner | *Notropis greenei* |
| White bass* | *Morone chrysops* |
| White crappie | *Pomoxis annularis* |
| White perch | *Morone americana* |
| White sucker | *Catostomus commersonii* |
| Yellow bullhead | *Ameiurus natalis* |
| Yellow bass | *Morone mississippiensis* |
| Yellow perch* | *Perca flavescens* |
